# Persistence of neutrophil extracellular traps and anticardiolipin auto‐antibodies in post‐acute phase COVID‐19 patients

**DOI:** 10.1002/jmv.28209

**Published:** 2022-10-31

**Authors:** Ekaterina Pisareva, Stephanie Badiou, Lucia Mihalovičová, Alexia Mirandola, Brice Pastor, Andrei Kudriavtsev, Marie Berger, Camille Roubille, Pierre Fesler, Kada Klouche, Jean‐Paul Cristol, Alain R. Thierry

**Affiliations:** ^1^ IRCM, Institut de Recherche en Cancérologie de Montpellier, INSERM U1194, Institut Régional du Cancer de Montpellier Université de Montpellier Montpellier France; ^2^ Department of Biochemistry and Hormonology, INSERM, CNRS, University Hospital Center of Montpellier University of Montpellier, PhyMedExp Montpellier France; ^3^ Faculty of Medicine, Institute of Molecular Biomedicine Comenius University Bratislava Slovakia; ^4^ Department of Internal Medicine, INSERM U1046, CNRS, Montpellier University Hospital, Montpellier, PhyMedExp University of Montpellier Montpellier France; ^5^ Intensive Care Medicine Department, INSERM, CNRS, Lapeyronie Hospital University Hospital of Montpellier, France, and PhyMedExp, University of Montpellier Montpellier France; ^6^ Montpellier Cancer Institute (ICM) Montpellier France

**Keywords:** anti‐cardiolipin, circulating DNA, COVID‐19, diagnosis, long COVID, neutrophil extracellular traps

## Abstract

In the early phase of the pandemic, we were among the first to postulate that neutrophil extracellular traps (NETs) play a key role in COVID‐19 pathogenesis. This exploratory prospective study based on 279 individuals showed that plasma levels of neutrophil elastase, myeloperoxidase and circulating DNA of nuclear and mitochondrial origins in nonsevere (NS), severe (S) and postacute phase (PAP) COVID‐19 patients were statistically different as compared to the levels in healthy individuals, and revealed the high diagnostic power of these NETs markers in respect to the disease severity. The diagnostic power of NE, MPO, and cir‐nDNA as determined by the Area Under Receiver Operating Curves (AUROC) was 0.95, 097, and 0.64; 0.99, 1.0, and 0.82; and 0.94, 1.0, and 0.93, in NS, S, and PAP patient subgroups, respectively. In addition, a significant fraction of NS, S as well as of PAP patients exhibited aCL IgM/IgG and anti‐B2GP IgM/IgG positivity. We first demonstrate persistence of these NETs markers in PAP patients and consequently of sustained innate immune response imbalance, and a prolonged low‐level pro‐thrombotic potential activity highlighting the need to monitor these markers in all COVID‐19 PAP individuals, to investigate postacute COVID‐19 pathogenesis following intensive care, and to better identify which medical resources will ensure complete patient recovery.

## 1 INTRODUCTION

COVID‐19 is associated with several clinical syndromes such as uncomplicated disease, asymptomatic forms, and symptomatic syndromes involving both nonsevere and severe pneumonia, acute respiratory distress syndrome (ARDS), and life‐threatening respiratory failure, as well as sepsis and septic shock with multivisceral failure syndrome.^1^, ^2^ There is now an accumulation of evidence which calls for the recognition of postacute COVID‐19 syndrome, characterized by long‐lasting mild or marked complications.^1^

In the early phase of the pandemic, we were among the first to postulate that neutrophil extracellular traps (NETs) play a key role in COVID‐19 pathogenesis, based on the numerous COVID‐19 biological and pathological features which can be seen as analogous with the double‐edged deleterious effects of NETs, as seen in other sterile and nonsterile pathologies.^3^, ^4^ The production of NETs (a process termed netosis) is a neutrophil function that plays an important role in the first line of innate immune defense. In this process, neutrophils are activated in response to infection and then release NETs, which are extensive structures composed of a scaffold of released chromatin decorated with granular proteins. By mechanically trapping microorganisms in the blood, these structures inhibit their dissemination, and employ the coagulation function to isolate those microorganisms in the circulation.^3^, ^5^ The inflammatory process is also in part triggered by circulating NETs by‐products, such as cir‐nDNA, cir‐mtDNA (acting as damage‐associated molecular patterns), histones, and granule proteins such as powerful catalytic enzymes such as neutrophil elastase (NE), or myeloperoxidase (MPO).^3^, ^5^ The resulting inflammatory processes and multiorgan damage can be characterized as complex diseases specifically associated with cytoxicity toward endothelial and epithelial cells, prothrombotic activity, and an abnormality of coagulation factors, resulting notably in systemic vascular permeability.^6^ Thus, exaggerated uncontrolled NETs formation may result in myocardial infarction, vasculitis, hemorrhage or systemic side effects within the circulation, and in multiple organ malfunction.^6,7^ Thus, NETopathies such as COVID‐19 may lead to an excessive concentration of neutrophils in lung vascularization, as well as elevated levels of interferon, lactate dehydrogenase (LDH), C‐reactive protein (CRP), pro‐inflammatory cytokines, and excessive levels of circulating fibrinogen, which together have the potential to induce respiration function failure to the extent of ARDS, as well as sepsis, thrombosis, and acute cardiac injury or indeed outright heart failure.^2^, ^3^, ^5^, ^6^

A few studies have reported the association of COVID‐19 with elevated plasma concentrations of MPO and NE, as well as of quantities of fragmented cirDNA,^8^, ^9^, ^10^ We recently demonstrated that NETs are a significant source of cirDNA of either nuclear origin in the circulation.^11^ Given that NETs and anticardiolipin (aCL) have been independently found to be associated with the occurrence of thrombosis in various diseases, our study concurrently assessed NET markers, cirDNA, and aCL levels, to investigate their association and their potential as COVID‐19 biomarkers. Towards this goal, we monitored the levels of these markers in COVID‐19 NS patients (*N* = 26), S patients (*N* = 44), and PAP subjects (*N* = 42). Data were compared with those obtained from control healthy individuals (HI) (*N* = 117). This is the first comprehensive study on the impact of NETs on COVID‐19 pathogenesis, in particular in individuals at least 6 months postrelease from ICU.

## 2 METHODS

**Figure 1:**
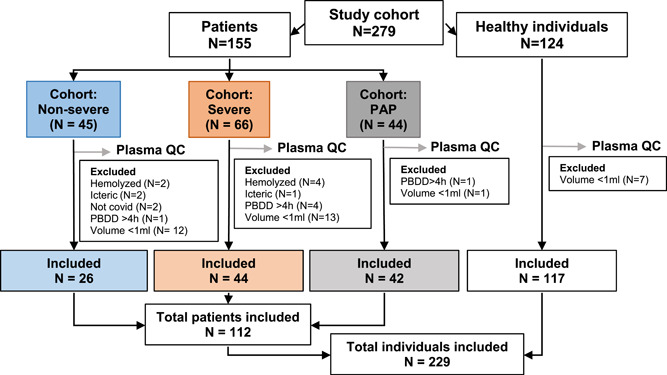
Study flow chart. PBDD: post blood draw delay; PAP: postacute phase.

Among the 279 plasma from COVID‐19 patients and HI individuals prospectively enrolled in the study, 229 (26S, 44 NS, 42 PAP, and 117 HI) passed the quality control step and were subsequently analyzed (Figure 1). All the COVID‐19 (NS and S) patients exhibited general COVID‐19 symptoms and characteristics as reported elsewhere^1^ (Supporting Information: Supplementary 1). We categorized patients as S versus NS depending on whether or not they met one or more of the following criteria: need for high‐flow nasal oxygen therapy (Optiflow; O2 > 15 L/min) or mechanical ventilation, transfer to the ICU during hospitalization, or occurrence of death (Supporting Information: Supplementary 1). The group of PAP patients consisted of 42 subjects previously hospitalized in an ICU, who were considered as S acute phase patients, and who were offered longitudinal monitoring for 6 months or more after discharge. At the time of blood collection, all PAP patients experienced at least one symptom listed as common in postacute sequelae of SARS‐Cov2 infection^12^ (Supporting Information: Table 5S). Patient characteristics, and comorbidities are described in Supporting Information: Tables 1‐2S. Biological analysis methods are described in Supporting Information: Supplementary 1, and data are reported in Supporting Information: Tables 3‐4S.

## 3 RESULTS AND DISCUSSION

### 3.1 Elevated NETs markers and cirDNA levels associated with disease severity

**Figure 2:**
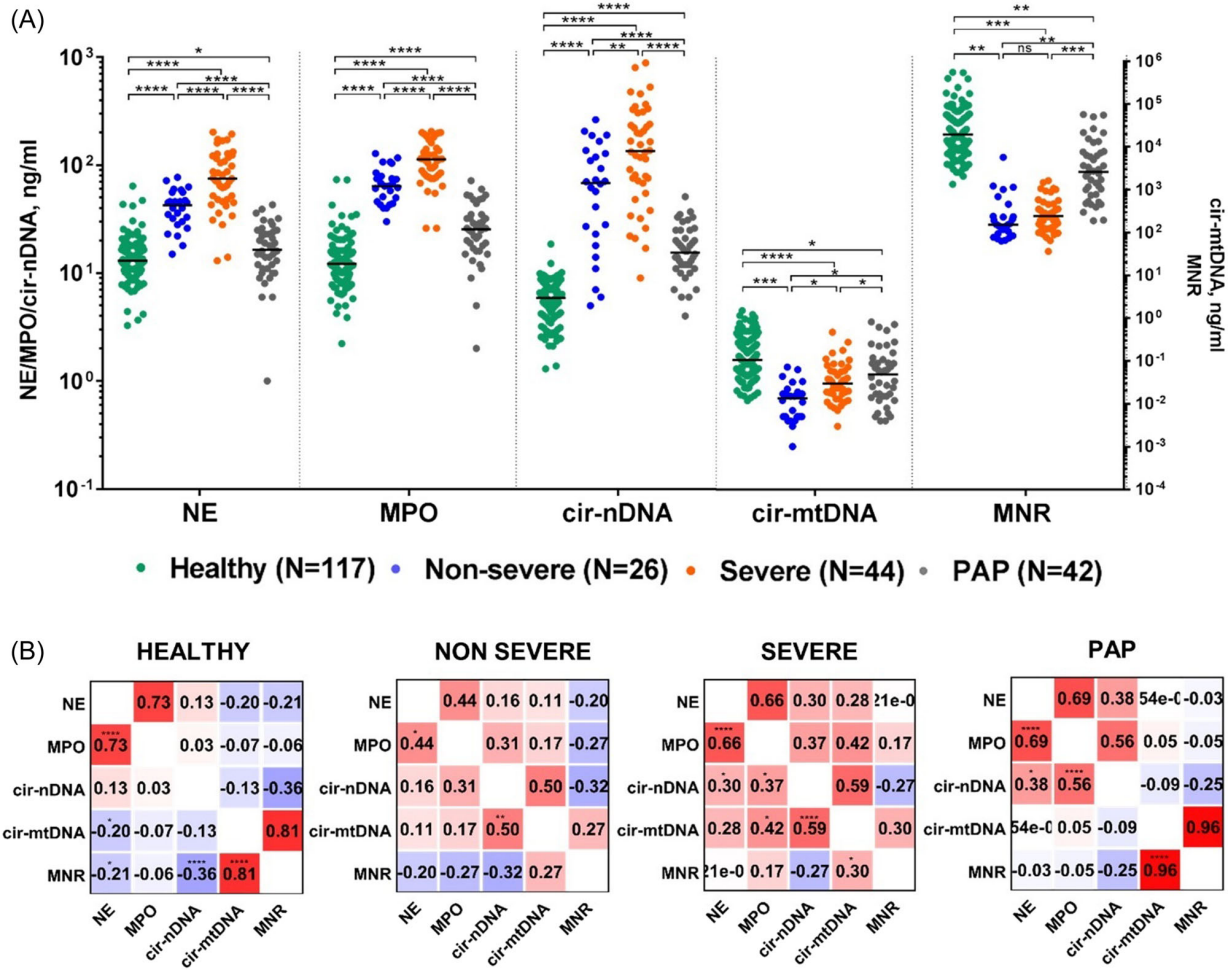
Nneutrophil extracellular traps (NETs)' association with COVID‐19 and postacute phase of COVID‐19. (A) Comparison of concentrations of NETs biomarkers (NE, MPO, cirDNA, cir‐mtDNA, and MNR) in healthy individuals (HI), nonsevere (NS), severe (S) and postacute phase (PAP) COVID‐19 patients. Lines represent median. *T*‐test was performed to compare the values of NETs biomarkers in COVID‐19 patients and HI. CirDNA: circulating cell‐free DNA; MPO, myeloperoxidase; NE, neutrophil elastase; cir‐mtDNA, circulating cell‐free DNA of mitochondrial origin; MNR, ratio of mitochondrial to nuclear circulating DNA concentration. Data obtained when combining NS and S patient cohorts (*N* = 70) are shown in Supporting Information: Figure 3S. (B) Correlation matrix of concentrations of NETs biomarkers (NE, MPO in ng/ml of plasma) with cirDNA markers (cirDNA, cir‐mtDNA in ng/ml plasma, MNR) in HI and NS, S, and PAP COVID‐19 patients. Heatmap manifests the strength of the relationship by Pearson's correlation analysis (red: positive correlation; blue: negative correlation). As shown in Supporting Information: Figure 2S the same observations are made when normalizing the concentration values with the neutrophil number suggesting that they are not directly due to the number of neutrophils. A probability of less than 0.05* was considered statistically significant; **p < 0.01, ***p < 0.001, ****p < 0.0001. Each dot represents the value of a single patient or a single healthy individual.

We observed that NE, MPO and cir‐nDNA concentrations in plasma were statistically significantly elevated in COVID‐19 NS and S patients compared to HI (Figure 2A, Supporting Information: Figures 2–3S and Tables 6‐9S). The highest values of these markers were found in the plasma of severe COVID‐19 patients, which showed significant differences from HI in the analysis of NE (74.6 ng/ml vs. 12.9 ng/ml, *p* < 0.000001), MPO (112.9 ng/ml vs. 12.2 ng/ml, *p* < 0.000001) and cirDNA (134.9 ng/ml vs. 5.9 ng/ml, *p* < 0.000001). The statistical differences found here between COVID patients and healthy subjects are higher than previously reported.^5^, ^8^, ^10^ Values of these markers in S patient plasma are statistically higher than in NS patients (Figure 2A; *p* < 0.0001 for both NE and MPO; and *p* < 0.01 for cir‐nDNA). When comparing S and HI cohorts, there is a 5.8, 9.3, and 22.9 fold‐increase of the median level of NE, MPO, and cir‐nDNA, respectively (Supporting Information: Table 10S).

In light of our previous studies,^13^ we also applied an index determined by the cir‐mtDNA/cir‐nDNA ratio (MNR, Supporting Information: Supplementary 1), which demonstrates a high capacity to differentiate cirDNA according to its origin. In this study, cir‐mtDNA, and MNR were significantly lower in COVID‐19 S and NS patients compared to HI (Figure 2A and Supporting Information: Figures 2–3S). There was no correlation between cir‐nDNA and NE/MPO concentrations in HI, while they correlated positively in COVID‐19 patients (Figure 2B). Cir‐mtDNA did not associate with cir‐nDNA in HI, and correlate positively in S and NS patients. MNR did not correlate or correlated weakly and negatively with cir‐nDNA, NE, and MPO in HI and NS patients, but did not correlate with NE and MPO in S and PAP patients. Note, the significant statistical MNR decrease we observed here in COVID‐19 patients might suggest compromised mitochondria‐nuclear coregulation, as speculated by Medini et al.^14^

Thus, our data confirmed observations we previously made in relation to metastatic colorectal cancer,^11^ namely that NETs protein biomarkers are associated with the generation of cirDNA, clearly demonstrating that NETs degradation in blood leads to chromatin fragmentation mostly resulting at the end to circulating mononucleosomes associated DNA. In addition, our present study confirmed both our own previous postulates and those of Barnes et al.,^4^ which clearly link the production of NETs in COVID‐19 patients and highlight the potential NETs key role in COVID‐19 pathogenesis. In addition to the release of excessive amounts of pro‐inflammatory cytokines, acute infection is associated with a high number of hyperactivated degranulating neutrophils.^15^ We postulate that cir‐nDNA is a strong additional marker of NETs in the COVID‐19 disease. The by‐products of NETs may be implicated in the pathogenesis of COVID‐19, with elastase notably playing a role in accelerating virus entry. As in numerous other NETopathies,^3^, ^4^, ^16^ those by‐products may also induce hypertension, thrombosis and vasculitis.^5^, ^7^, ^15^, ^17^ In relative concordance with our observations which differentiated NS and S cohorts using NET markers levels, 0/26 and 6/44 (13.6%) patients experienced GI bleeding in NS and S patients, respectively. In addition, levels of ferritin, creatine kinase (CK), lactate dehydrogenase (LDH), CRP, polymorphonucleic cell % (PNN), and d‐dimers on the one hand, and calcium, lymphocytes to neutrophils ratio (LNR) on the other hand, showed positive and negative associations, respectively, with NE, MPO, and cir‐nDNA levels in the NS and S cohorts (Supporting Information: Figure 1S). All those markers are considered as nonspecific prognostic biomarkers of COVID‐19.^18^ We speculate that SARS‐CoV2 may activate an innate immune response, resulting in an uncontrolled formation of NETs, and inducing multiorgan failure in high‐risk individuals.

### 3.2 Anti‐cardiolipin auto‐antibodies are often present in COVID‐19 patients and associated with NETs

**Figure 3:**
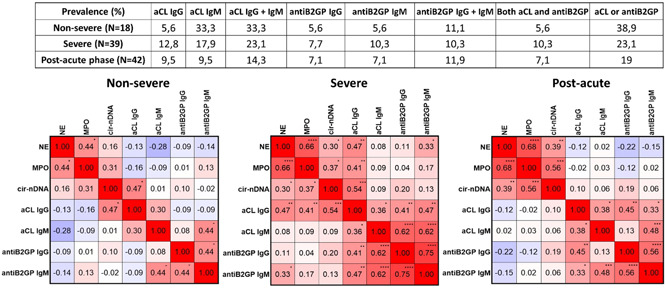
Presence of the anti‐cardiolipin autoantibodies (anticardiolipin [aCL], IgG/IgM) and Beta‐2‐glycoprotein I antibodies (anti‐B2GPI, IgG/IgM) in nonsevere (NS), S, and postacute phase (PAP) COVID‐19 patients. Positive patient number in these cohorts are indicated in Supporting Information: Figure 7S. Measurement of aCL and the B2GPI, IgG/IgM in plasma subjects was performed using an ELISA kit. B2GP binds anionic phospholipids and is considered to be the predominant antigen in anti‐phospholipid syndrome (APS), with anti‐B2GP recognized in the laboratory criteria for APS diagnosis. An increased prevalence of anti‐B2GP outside of APS has been reported in several infections, as well as in a variety of other disorders (Supporting Information: Supplementary 1). The rather low aCL test specificity is improved by the use of the ELISA as a confirmatory, specific test for B2GPI antibodies, usually being performed after a positive screening test result for aCL. Quantitative assessment of aCL antibodies was considered for IgG or IgM ≥ 3 UA/ml (upon the 99th percentile in healthy controls using the Orgentec Diagnostika Elisa kit). Quantitative assessment of anti‐B2GP antibodies was considered for IgG ≥ 4 UA/ml and IgG ≥ 5 UA/ml (upon the 90th percentile). Note, IgG and IgM of aCL and anti‐B2GP are statistically associated in several S and PAP subjects validating the use of the cutoffs. A probability of less than 0.05* was considered statistically significant; **p < 0.01, ***p < 0.001, ****p < 0.0001.

We observed aCL and Beta‐2 glycoprotein antibody (anti‐B2GP) presence in a significant fraction of NS and S patients (38.9% IgM and IgG, and IgM and IgG 23.1%, respectively). aCL correlation with anti‐B2GP is clearly apparent (Figure 3), as has previously been observed for various diseases, such as the anti‐phospholipid syndrome (APS).^19^ The aCL prevalence levels we determined correspond to those reported in several very recent reports on COVID‐19.^20^, ^21^, ^22^ Associations between both antibodies as well as between NETs markers and both antibodies were observed in the S group, and to a lesser extent in the NS group (Figure 3). Although the detection of anti‐phospholipid antibody (aPL) such as aCL has shown potential as a strategy in preventing thrombosis, the direct or indirect role of aPL in COVID‐19 thrombophilic coagulopathy has yet to be fully understood.^6^ Shi et al.^23^ spectulate that endothelial cells may be activated by aPL, which may in turn induce a pro‐adhesive phenotype. That said, the contribution of neutrophils and NETs to APS pathophysiology is nonetheless evident.^24^ The link has also been established between exacerbated NETs formation and APS in multiple auto‐immune and nonauto‐immune pathologies (including lupus) which exhibit raised aCL levels.^3^ Note, the progressive expansion of the intima by cell proliferation, leading to organ damage, characterizes occlusive vasculopathy in APS.^6^ In addition, thrombotic complications have been reported to associate with aCL positivity in some cases of a variety of viral infections.^19^ NETs and thrombi were found to colocalize in COVID‐19^9^; more precisely, cirDNA and MPO activity were associated in patients with thrombotic micro‐angiopathies.^16^

### 3.3 NETs and cirDNA markers showed high diagnostic capacity

**Figure 4:**
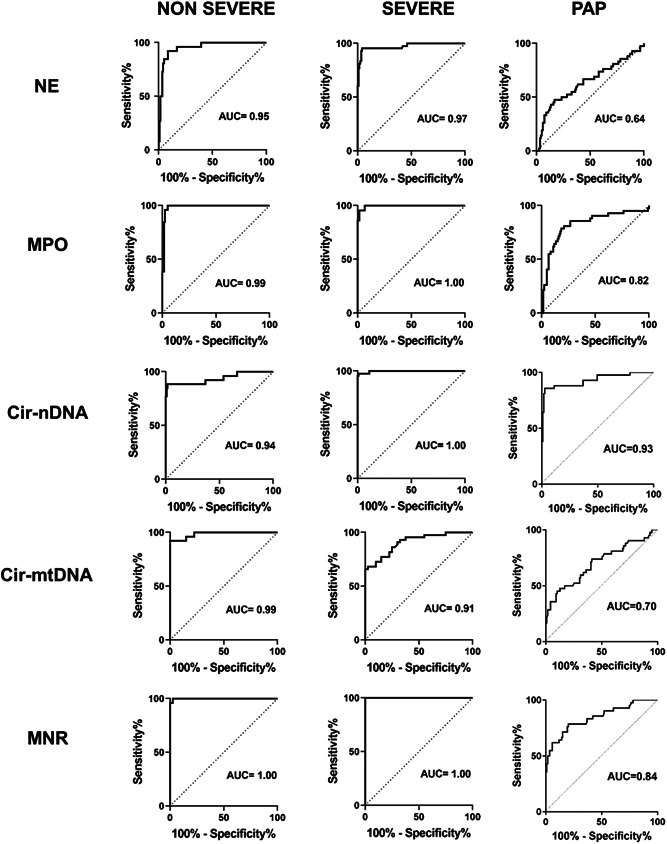
Performance characteristics of the neutrophil extracellular traps (NETs) biomarkers for COVID‐19. Receiver operating curves (ROC) curves for neutrophil elastase (NE), myeloperoxidase (MPO), cir‐nDNA, cir‐mtDNA concentrations and MNR between healthy individuals and COVID‐19 patients (nonsevere [NS], S, and postacute phase [PAP]). ROC curves of these markers in combining both NS and S COVID‐19 patient cohorts versus HI (AUC of 0.97, 0.99, 0.98, and 1.0 for NE, MPO, cir‐nDNA, and MNR, respectively), and in differentiating NS and S (0.81, 0.81, 0.72, and 0.60 for NE, MPO, cir‐nDNA, and MNR, respectively) are shown in Supporting Information: Figure 4S and 5S, respectively. The improved diagnostic power (AUC of 0.98 and 0.99 in NS and S cohorts, respectively) by combining NE and MPO concentrations is shown in Supporting Information: Figure 6S. AUC, area under curve; cirDNA, circulating cell‐free DNA; cir‐mtDNA, circulating cell‐free DNA of mitochondrial origin; MNR, ratio of mitochondrial to nuclear circulating DNA concentration; MPO, myeloperoxidase; NE, neutrophil elastase; ROC, receiver operating characteristics.

As determined from receiver operating characteristics (ROC) curves (Figure 4), NE (area under curve AUC 0.95, 0.97, and 0.64), MPO (0.99, 1.00, and 0.82) and cir‐nDNA (0.94, 1.00, and 0.93) showed high levels of diagnostic capacity for NS, S, and PAP individuals, respectively, as compared with HI. When comparing a combination of both NS and S COVID‐19 patient cohorts with HI, we observed AUC of 0.97, 0.99, 0.98, and 1.0 for NE, MPO, cir‐nDNA, and MNR, respectively (Supporting Information: Figure 4S); when differentiating NS and S, we observed AUC of 0.81, 0.81, 0.72, and 0.60 for NE, MPO, cir‐nDNA and MNR, respectively (Supporting Information: Figure 5S).

### 3.4 High levels of NETs markers and cirDNA persisted in postacute phase patients

While the concentrations of NE, MPO, and cir‐nDNA were lower in PAP patients as compared to NS and S, they were statistically higher than in HI (PAP vs. HI: NE: 16.8 vs. 12.9 ng/µl; MPO: 25.7 vs. 12.2 ng/µl; cir‐nDNA: 15.2 vs. 5.9 ng/µl) (Figure 2A). NETs formation in PAP patients may appear frequent, given that 90.5% of PAP patients showed NE, MPO, and cir‐nDNA levels which were above the healthy median levels, as compared with 100% in NS and S patients (Supporting Information: Tables 6‐8S). Note, fibrinogen and thrombocytes coagulation factors are statistically more elevated in NS and S COVID‐19 patients while a bleeding marker such as hemoglobin is significantly and statistically more elevated in PAP subjects as compared to NS, S patients and healthy individuals (Supporting Information: Tables 11S). CRP is one of the solid markers of systemic inflammation in COVID‐19^18^ and has been identified as an early detector of disease severity,^25^ an independent predictor of death,^26^ and a suitable biomarker to guide therapy in patients with COVID‐19.^27^ Whereas NETs markers' association with disease severity is in line with CRP positive correlation with NETs markers in NS and S patients, no correlation was found between NETs markers and CRP in PAP patients (Supporting Information: Figure 1S). In contrast to CRP normalization in 80%–90% of PAP patients due to the subsidence of systemic inflammation,^28^, ^29^ NETs markers have a potential clinical utility in the postacute disease (“long COVID‐19”). Prospective studies are necessary to enable these data to be translated into practical clinical applications, which could include prognostics, patient management, and treatment guidance. There was also a difference in the cir‐mtDNA, and MNR values of HI and PAP subjects (Figure 2A). Note, whereas there was a clear correlation between cir‐nDNA and NE/MPO concentrations in PAP patients, MNR and cir‐mtDNA were not associated with NE, MPO, and cir‐nDNA in PAP patients (Figure 1B). While their prevalence in NS patients did not correlate with NETs markers, in contrast to S patients, aCL and aB2GP were detected in 19.1% of PAP patients (Figure 3). Although the presence of these two auto‐antibodies was clearly detected in several patients (7/26, 9/44, and 8/42 in NS, S and PAP respectively), with a significant prevalence, the fact that the positive patient number was rather low means that their prevalence value should nonetheless be treated with caution.

## 4 CONCLUDING REMARKS

This study is the first to reveal that NETs and aCL production may be sustained for 6 months or more postacute infection. All PAP subjects we studied experienced at least one symptom listed among those in postacute sequelae of SARS‐Cov2 infection syndrome.^12^ Our observational study is limited by the number of PAP patients studied, and by the large scope of symptoms considered, which included general symptoms, neurological, cardiopulmonary, ORL, musculoskeletal, and gastrointestinal disorders of varying severity. This precluded observation of their specific association with NETs and cirDNA markers, as well as with aCL. In addition, the PAP patients studied may have had symptoms of different origins: (i), being secondary to COVID‐19–induced damage to the lung, heart, skeletal muscle, kidneys, or brain; (ii) persistent debilitating symptoms (post‐COVID syndrome or “long COVID‐19” illness) in patients with no apparent organ damage; or (iii), symptoms originating in both co‐occurring clinical conditions.^1^, ^12^, ^30^ Nonetheless, in light of the statistical difference found between NS, S, and PAP patients versus HI, we postulate that uncontrolled NETosis activation resulting from SARS‐CoV2 infection may be sustained by a feedback loop resulting from systemic NETs by‐products release as we previously speculated.^3^ Active investigation is urgently needed to understand the nature of this serious and long‐lasting phenomenon, and then to develop suitable therapy towards complete recovery. The markers examined in this study could be employed as biomarkers until their usefulness to clinicians in decision‐making concerning treatment is proven. Nonetheless, they showed a very high diagnostic power, exhibited association with disease severity and with postacute phase, and may contribute significantly to achieving this public health objective.

## AUTHOR CONTRIBUTIONS

Alain R. Thierry and Ekaterina Pisareva designed the study. Ekaterina Pisareva and Alain R. Thierry developed the methodology. Stephanie Badiou, Jean‐Paul Cristol, Ekaterina Pisareva, Lucia Mihalovičová, Brice Pastor, Andrei Kudriavtsev, and Alexia Mirandola did the experiments on NETs markers. Ekaterina Pisareva, Lucia Mihalovičová, and Alexia Mirandola did the statistical analyses. Stephanie Badiou, Jean‐Paul Cristol, Pierre Fesler, Kada Klouche, Marie Berger, and Camille Roubille provided the plasma samples of COVID‐19 patients and PAP individuals, and all clinical laboratory data. Jean‐Paul Cristol and Stephanie Badiou obtained the ethics committee approval. Ekaterina Pisareva and Alain R. Thierry analyzed the data and prepared the manuscript. All of the authors (Ekaterina Pisareva, Lucia Mihalovičová, Brice Pastor, Andrei Kudriavtsev, Alexia Mirandola, Pierre Fesler, Kada Klouche, Marie Berger, Camille Roubille, Jean‐Paul Cristol, and Alain R. Thierry) discussed the results and approved the manuscript.

## CONFLICT OF INTEREST

The authors declare no conflict of interest.

## ETHICS STATEMENT

This observational prospective study was approved by the local ethics committee (ID_202100715). All enrolled patients approved written patient statement.

## Supporting information

Supplementary information.

Supplementary information.

## Data Availability

The data that support the findings of this study are available from the corresponding author upon reasonable request. Data are available upon request to the authors Stephanie Badiou, Jean‐Paul Cristol, and Alain R. Thierry had full access to all the data in the study and takes responsibility for the integrity of the data and the accuracy of the data analysis. Data will be made available upon request.
