# Supplementary material for: Persistence of neutrophil extracellular traps and anticardiolipin auto‐antibodies in post‐acute phase COVID‐19 patients
Source: J Med Virol. 2022 Oct 31;95(1):e28209. doi: 10.1002/jmv.28209 (PMC9874393; doi:10.1002/jmv.28209)
Supplement: Supplementary file 1 — Supplementary information. [file JMV-95-e28209-s001.pdf]

Table 1S. Characteristics of hospitalized patients at admittance

	Non-severe (n=26)<br>Median [min-max]	Severe (n=44)<br>Median [min-max]	p-value
Age (years)	67 [39-83]	70 [18-81]	NS
Sex M/F; %M	13/13; 50%	31/13; 70%	NS
BMI	26 [22-40]	28 [18-43]	NS
Obesity(%)	8 (31)	13 (30)	NS
Diabetes (%)	17 (65)	14 (32)	<0.05
Hypertension (%)	19 (73)	21 (48)	<0.05
Cardiovascular disease (%)	10 (38)	13 (30)	NS
Dyslipidemia (%)	13 (50)	12 (27)	NS
CKD (%)	4 (15)	3 (7)	NA
COPD (%)	2 (8)	2 (5)	NA
Current smoking (%)	2 (8)	1 (2)	NA
Cancer (%)	1 (4)	2 (5)	NA
Chronic immunosuppression (%)	4 (15)	1 (2)	NA
Transplantation(%)	3 (12)	1 (2)	NA

NS: no significative, NA: not applicable (n<5)

Table 2S. Characteristics of PAP individuals (n=42) at consultation for long term follow-up

PAP	median [min-max]	n (%)
Age (years),	66 [31-78]	
Sex M		39 (93)
BMI, median [min-max]	29 [21-41]	
Obesity, n (%)		20 (48)
Diabetes, n (%)		12 (29)
Hypertension, n (%)		17 (40)
Pulmonary disease, n (%)		7 (17)
CKD, n (%)		7 (17)
Autoimmune disease, n (%)		1 (2.4)
Current smoking, n (%)		2 (5)
History of cancer, n (%)		9 (21)
Chronic immunosuppression n (%)		0 (0)
Dyslipidemia, n (%)		17 (40)
Transplantation, n (%)		0 (0)

Table 3S. Biological data of hospitalized patients at admittance

	Non-severe (n=26) Median [min-max]		Severe (n=44) Median [min-max]		p-value
Creatinine (µmol/l)	67	[41-182]	70	[39-842]	NS
eDFG (ml/min/1,73m²)	90	[25-116]	90	[4-117]	NS
Uric acid (µmol/L)	318	[124-608]	243	[92-702]	NS
Albumin (g/l)	34	[28-44]	32	[21-41]	<0.05
Corrected calcemia (mmol/l)	2.39	[2.13-2.84]	2.33	[2.06-2.64]	<0.05
Phosphate (mmol/l	1.08	[0.82-1.84]	0.98	[0.36-2.25]	NS
Ferritine (µg/L)	942	[132- 6975]	1070	[175- 9311]	NS
Total Bilirubin (µmol/L)	7	[2-12]	65	[3-36]	NS
ASAT (UI/L)	29	[13- 146]	45	[16-574]	NS
ALAT (UI/L)	29	[9-234]	29	[9-488]	NS
ALP(UI/L)	68	[43-286]	75	[37- 371]	NS
GGT(UI/L)	51	[12-309]	66	[10- 460]	NS
LDH	291	[94-402]	427	[161-2374]	<0.01
CRP (mg/L)	44	[4 -172]	94	[6 -450]	<0.001
PCT (ng/l)	0.1	[0.05-1.64]	0.21	[0.02-133]	NS
HbA1c (%)	7.0	[5.5 -10.4]	6.6	[5.5 -10.3]	NS
Hemoglobin (g/L)	134	[109-156]	129	[98-166]	NS
Leukocytes (10^9/L)	5.8	[1.4-10.9]	7.7	[2.8-25.5]	<0.01
Neutrophil (10^9/L)	4.4	[0.7-8.6]	6.3	[1.4-22.9]	<0.01
Lymphocytes (10^9/L)	1.0	[0.4-2.0]	0.9	[0.3-2.3]	NS
Thrombocytes (10^9/L)	251	[148-543]	248	[113-655]	NS
Fibrinogen (g/L)	5.1	[2.9-8.0]	5.9	[0.8-9.1]	NS
D-Dimer (ng/mL)	751	[372-2583]	1009	[309-3649]	NS
a-CL IgM (UI/L)	2	[1-15]	1	[1-20]	NS
a-CL IgG (UI/L)	1	[1-3]	1	[1-11]	NS
a-B2GP1 IgM (UI/L)	1	[1-11]	1	[1-30]	NS
a-B2GP1 IgG (UI/L)	1	[1-6]	1	[1-7]	NS
a-CL (> 40), n (%)	0 (0)		0 (0)		NS
a-B2GPI (> 40), n (%)	0 (0)		0 (0)		NS

Table 4S. Biological data of PAP population at consultation for long term follow-up (n=42)

	Median [min-max]	Outer the reference range, n (%)	Reference range
Creatinine (µmol/l)	85 [38-138]	9 (21)	M: 59-104 F: 45-84
eDFG (ml/min/1,73m²)	85 [44-120]	7 (17)	>=60
Albumin (g/l)	43 [37-52]	0 (0)	35-52
Ferritine (µg/L)	177 [23-1797]	9 (21)	M:30-400 F: 20-200
Total bilirubin (µmol/L)	8 [4-53]	1 (2)	<21
ASAT (UI/L)	20 [12-112]	1 (2)	M: <40 F:< 32
ALAT (UI/L)	18 [7-215]	5 (12)	M: <41 F:< 33
ALP(UI/L)	67 [31-143]	1 (2)	M:<130 F:<105
GGT(UI/L)	29 [11-145]	8 (19)	M: <60 F:< 40
CRP (mg/L)	2.1 [0.3-47.9]	8 (19)	<5
HbA1c (%)	6.0 [4.7-10]	29 (69)	> 5.7
Hemoglobin (g/L)	14.8 [12.5-17.6]	3 (6)	M:130-180 F:120-160
Leukocytes (10^9/L)	5.9 [2.4-11.0]	7 (17)	4.0-10.0
Neutrophil (10^9/L)	3.2 [1.1-5.3]	4 (10)	2.0-7.5
Lymphocytes (10^9/L)	1.9 [0.6-6.6]	4 (10)	1.0-4.0
Thrombocytes (10^9/L)	210 [139-392]	3 (7)	150-400
Fibrinogen (g/L)	3.4 [2.3-4.4]	7 (17)	1.9-4.0
Lupus anticoagulant, n (%)	3 (7)		-
a-CL IgM (UI/L)	1.0 [1.0-6.0]	0 (0)	<7
a-CL IgG (UI/L)	1.0 [1.0-6.0]	0 (0)	<10
a-B2GP1 IgM (UI/L	1.0 [1.0-7.0]	2 (5)	<5
a-B2GP1 IgG (UI/L	1.0 [1.0-20]	2 (5)	<5
a-CL (> 40) n (%)	0 (0)		
a-B2GPI (> 40), n (%)	0 (0)		

Table 5S. Post-COVID events and manifestations in the PAP population (n=42)

	All, n (%)	Resolutive, n (%)
**General manifestations**	**36 (85.7)**	
Asthenia	32 (76.2)	11 (26.2)
Loss of weight (after discharge)	7 (16.7)	
Fever/Sweating/ Chills	1 (2.4)	
**Neurological manifestations**	**20 (47.6)**	
Headache	2 (4.8)	
Loss of memory	5 (11.9)	1 (2.4)
Attention disorders	8 (19.0)	1 (2.4)
Sleep disorders	9 (21.4)	
Depression	7 (16.7)	
**Cardio-thoracic symptoms**	**23 (54.8)**	
Dyspnea	18 (42.9)	2 (4.8)
Chest pain	8 (19.0)	2 (4.8)
Palpitation	3 (7.1)	1 (2.4)
Cough	3 (7.1)	
**ENT disorders**	**6 (14.3)**	
Agueusia	2 (4.8)	1 (2.4)
Anosmia	6 (14.3)	2 (4.8)
**Musculoskeletal disorders**	**30 (71.4)**	
Muscle weakness	24 (57.1)	19 (45.2)
Arthalgia	16 (38.1)	2 (4.8)
**Gastro-intestinal disorders**	**10 (23.8)**	
Gastro-intestinal bleeding	2 (4.8)	2 (4.8)
Constipation	5 (11.9)	
Biliary tract disease	3 (7.1)	
**Abbreviations :** ENT : Ears Nose Throat		

Table 6S. Values of NETs biomarkers for non-severe COVID-19 patients

Cohort	Sample	NE [ng/ml]	MPO [ng/ml]	cir-nDNA [ng/ml]	cir-mtDNA [ng/ml]	MNR
NON-SEVERE (N=26)	M09	60.1	106.9	110	0.016	1.44E+02
	M15	32.7	50.1	57	0.007	1.31E+02
	M18	44.6	74.3	264	0.063	2.35E+02
	M35	14.6	30.2	5	0.003	6.94E+02
	M39	34.9	42.5	7	0.001	8.41E+01
	M40	45.9	104.4	93	0.019	2.06E+02
	M41	28.1	75.2	128	0.012	9.45E+01
	M45	56.3	117.2	188	0.044	2.30E+02
	M52	22.5	45.9	11	0.005	4.64E+02
	M11	25.9	85.3	62	0.004	6.37E+01
	M14	40.0	79.4	28	0.004	1.52E+02
	M53	62.6	57.5	73	0.005	7.23E+01
	M55	77.1	61.7	14	0.016	1.15E+03
	M65	31.7	50.5	206	0.032	1.55E+02
	M30	46.0	43.1	67	0.005	7.46E+01
	M69	56.3	43.9	190	0.015	7.75E+01
	M76	45.1	60.9	70	0.072	1.02E+03
	M81	40.4	67.3	27	0.004	1.63E+02
	M51	36.2	107.3	137	0.015	1.06E+02
	M61	22.5	39.5	6	0.033	5.76E+03
	M17	72.4	127.9	119	0.017	1.39E+02
	M25	59.2	64.0	41	0.005	1.18E+02
	M50	18.1	68.2	71	0.017	2.41E+02
	M54	29.8	39.9	166	0.011	6.66E+01
	M 60	45.6	74.9	23	0.005	1.94E+02
	M 68	46.7	64.5	18	0.022	1.22E+03
	**MEAN**	**42.1**	**68.6**	**83.9**	**0.017**	**5.02E+02**
	**MEDIAN**	**42.5**	**64.2**	**68.6**	**0.013**	**1.54E+02**
	**SD**	**16.3**	**26.2**	**72.0**	**0.018**	**1.12E+03**
Healthy (N=117)	**MEAN**	**14.6**	**14.3**	**6.1**	**0.405**	**9.27E+04**
	**MEDIAN**	**12.9**	**12.2**	**5.9**	**0.277**	**5.65E+04**
	**SD**	**8.3**	**10.1**	**2.6**	**0.373**	**1.11E+05**

Legend: Manifestations of possible NETs formation in NS, S and PAP cohorts appear frequent, given that 100%, 100% and 90.5% of plasma showed NE, MPO and cir-nDNA levels which were above the healthy median level, and given that 92.4%, 100% and 71% of plasma showed at least two out of the three markers at a level more than double the healthy median level, respectively.

Table 7S. Values of NETs biomarkers for severe COVID-19 patients

Cohort	Sample	NE [ng/ml]	MPO [ng/ml]	cir-nDNA [ng/ml]	cir-mtDNA [ng/ml]	MNR
SEVERE (N=44)	R03	108.2	184.9	529	0.106	1.98E+02
	R04	126.7	181.2	458	0.473	1.02E+03
	R06	86.7	98.3	334	0.154	4.56E+02
	R08	71.7	83.4	312	0.178	5.65E+02
	R09	31.2	112.9	196	0.059	2.95E+02
	R11	44.7	55.2	76	0.013	1.65E+02
	R12	46.0	56.7	76	0.008	9.91E+01
	R15	28.1	113.9	305	0.037	1.21E+02
	R16	194.3	111.1	114	0.022	1.94E+02
	R19	202.0	194.0	239	0.036	1.48E+02
	R20	168.5	172.1	885	0.112	1.25E+02
	R21	70.9	130.6	55	0.011	1.89E+02
	R32	41.7	160.6	165	0.046	2.79E+02
	R29	63.3	125.2	155	0.017	1.07E+02
	R33	97.8	115.4	366	0.086	2.32E+02
	R35	85.3	94.3	199	0.027	1.36E+02
	R37	74.6	93.9	207	0.024	1.13E+02
	R38	115.2	85.1	328	0.012	3.63E+01
	R39	128.5	75.3	21	0.003	1.33E+02
	R42	87.4	63.6	137	0.040	2.90E+02
	R44	48.3	88.9	22	0.007	3.30E+02
	R49	43.4	77.0	72	0.021	2.94E+02
	R51	64.8	115.5	476	0.037	7.64E+01
	R53	36.4	109.6	123	0.032	2.57E+02
	R55	132.7	193.9	800	0.276	3.42E+02
	R56	48.0	81.0	349	0.056	1.58E+02
	R01	76.5	136.0	26	0.024	8.87E+02
	R13	159.9	205.1	81	0.038	4.66E+02
	R22	51.8	105.3	38	0.056	1.48E+03
	R23	124.4	140.8	68	0.013	1.93E+02
	R24	122.1	116.4	91	0.033	3.58E+02
	R25	172.7	143.7	226	0.072	3.16E+02
	R26	172.3	199.8	79	0.086	1.08E+03
	R36	117.8	199.0	48	0.079	1.63E+03
	R46	104.5	199.8	167	0.059	3.51E+02
	M12	62.3	68.3	234	0.023	9.70E+01
	M21	14.5	26.1	17	0.019	1.07E+03
	M38	33.9	74.8	118	0.011	9.33E+01
	M44	13.3	26.0	9	0.009	9.34E+02
	M49			32	0.019	5.87E+02
	M56	51.7	76.9	34	0.018	5.19E+02
	M74	63.6	114.4	221	0.020	8.93E+01
	M82	81.1	125.2	133	0.017	1.26E+02
	M83	70.1	79.0	132	0.009	6.53E+01
	**MEAN**	**86.9**	**116.5**	**199.0**	**0.057**	**3.80E+02**
	**MEDIAN**	**74.6**	**112.9**	**134.9**	**0.030**	**2.44E+02**
	**SD**	**49.0**	**48.5**	**194.3**	**0.083**	**3.82E+02**
Healthy (N=117)	**MEAN**	**14.6**	**14.3**	**6.1**	**0.405**	**9.27E+04**
	**MEDIAN**	**12.9**	**12.2**	**5.9**	**0.277**	**5.65E+04**
	**SD**	**8.3**	**10.1**	**2.6**	**0.373**	**1.11E+05**

Table 8S. Values of NETs biomarkers for post-acute phase (PAP) COVID-19 cohort

Cohort	Sample	NE [ng/ml]	MPO [ng/ml]	cir-nDNA [ng/ml]	cir-mtDNA [ng/ml]	MNR
PAP (N=42)	M01	22.9	29.5	16	0.027	1.73E+03
	M02	25.8	29.0	20	0.055	2.72E+03
	M07	22.1	29.0	25	0.062	2.49E+03
	M13	32.1	43.6	35	0.119	3.38E+03
	M23	24.7	60.1	20	0.042	2.09E+03
	M29	38.9	53.0	14	0.091	6.34E+03
	M04	19.5	31.6	12	0.088	7.22E+03
	M42	23.4	48.3	14	0.108	7.65E+03
	M47	36.1	25.3	9	0.034	3.62E+03
	M62	23.6	17.9	6	0.017	2.98E+03
	M77	24.5	35.4	15	0.015	1.04E+03
	M79	18.7	17.1	4	0.024	5.99E+03
	PA01	14.8	12.6	13	0.031	2.40E+03
	PA02	11.4	10.8	10	0.224	2.16E+04
	PA03	30.4	20.4	37	0.064	1.71E+03
	PA04	15.9	21.3	17	0.025	1.45E+03
	PA05	27.6	31.5	11	0.616	5.58E+04
	PA06	24.8	51.7	31	0.155	5.00E+03
	PA07	19.1	21.6	16	0.815	5.09E+04
	PA08	30.8	52.7	30	0.236	7.83E+03
	PA09	20.8	25.9	6	0.090	1.56E+04
	PA10	10.3	31.9	26	0.012	4.54E+02
	PA11	5.8	25.4	19	0.519	2.67E+04
	PA12	10.7	18.8	12	0.716	5.74E+04
	PA13	10.0	23.7	18	0.069	3.69E+03
	PA14	17.1	48.3	33	0.015	4.59E+02
	PA15	13.7	26.8	12	0.074	6.21E+03
	PA16	18.1	49.5	18	0.251	1.35E+04
	PA17	11.8	8.6	11	0.004	3.95E+02
	PA18	15.4	16.0	33	0.017	5.16E+02
	PA19	16.4	25.9	19	0.014	7.45E+02
	PA20	8.7	21.7	13	0.279	2.15E+04
	PA21	5.8	12.5	14	0.017	1.23E+03
	PA22	9.9	19.4	21	0.004	1.96E+02
	PA23	13.6	16.3	12	0.012	1.01E+03
	PA24	12.6	35.3	25	0.008	3.00E+02
	PA25	0.6	1.6	7	0.005	7.76E+02
	PA26	14.9	25.9	7	0.088	1.28E+04
	PA27	8.1	15.1	10	0.006	5.34E+02
	PA28	11.9	4.7	12	0.006	4.81E+02
	PA29	42.9	71.6	51	0.060	1.17E+03
	PA30	8.9	15.0	25	0.005	1.90E+02
	**MEAN**	**18.5**	**28.1**	**18.0**	**0.122**	**8.57E+03**
	**MEDIAN**	**16.8**	**25.7**	**15.2**	**0.048**	**2.61E+03**
	**SD**	**9.4**	**15.6**	**10.0**	**0.195**	**1.44E+04**
**Healthy (N=117)**	**MEAN**	**14.6**	**14.3**	**6.1**	**0.405**	**9.27E+04**
	**MEDIAN**	**12.9**	**12.2**	**5.9**	**0.277**	**5.65E+04**
	**SD**	**8.3**	**10.1**	**2.6**	**0.373**	**1.11E+05**

Table 9S. Summary data of NETs biomarkers for COVID-19 patients and PAP subjects.

	Non-severe (N=26)			Severe (N=44)			PAP (N=42)		
	MEAN	MEDIAN	SD	MEAN	MEDIAN	SD	MEAN	MEDIAN	SD
**NE [ng/ml]**	42.1	42.5	16.3	86.9	74.6	49.0	18.5	16.8	9.4
**MPO [ng/ml]**	68.6	64.2	26.2	116.5	112.9	48.5	28.1	25.7	15.6
**cir-nDNA [ng/ml]**	83.9	68.6	72.0	199.0	134.9	194.3	18.0	15.2	10.0
**cir-mtDNA [ng/ml]**	0.017	0.013	0.018	0.057	0.030	0.083	0.122	0.048	0.195
**MNR**	5.02E+02	1.54E+02	1.12E+03	3.80E+02	2.44E+02	3.82E+02	8.57E+03	2.61E+03	1.44E+04

Table 10S. Comparison of NETs biomarkers in COVID-19 patients (S: severe; NS: non-severe), post-acute phase (PAP) and healthy individuals (HI)

(+) X fold higher

(-) X fold lower

NET marker	S vs HI	NS vs HI	PAP vs HI	S vs NS
NE	(+) 5.8	(+) 3.3	(+) 1.3	(+) 1.8
MPO	(+) 9.3	(+) 5.3	(+) 2.1	(+) 1.8
cir-nDNA	(+) 22.9	(+) 11.6	(+) 2.6	(+) 2
cir-mtDNA	(-) 9.2	(-) 21.3	(-) 5.8	(+) 2.3
MNR	(-) 231.6	(-) 366.9	(-) 21.6	(+) 1.6

Table 11S. Comparison of Fibrinogen, Thrombocytes, and Hemoglobin plasma concentration in COVID-19 patients (S: severe; NS: non-severe) and Post-Acute hase individuals (PAP).

Fibrinogen is highly elevated in NS and S patients while being sliglthly elevated in PAP individuals. Thrombocytes number is moderately elevated in NS and S patients while being normalized in PAP individuals. Hemoglobin concentration is within the reference range in NS, very slighly elevated in S patients and statistically more elevated in PAP individuals. Data seem to show that Fibrinogen and Thrombocytes coagulation factors are statistically more elevated in NS and S COVID-19 patients while a bleeding marker such as Hemoglobin is statistically more elevated in PAP subjects.

Fibrinogen

Number of subjects outer the reference range (>4.0 g/L):
18 NS (69%)
39 S (89%)
7 PAP (17%)

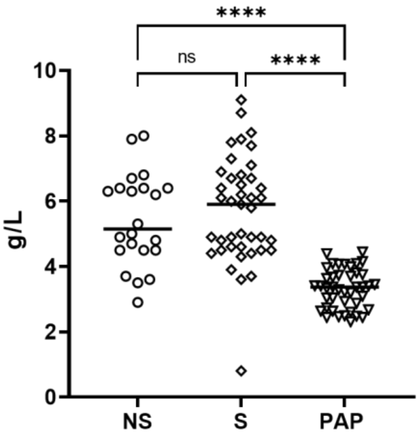

Thrombocytes

Number of subjects outer the reference range (>400 10^9/L):
3 NS (12%)
9 S (20%)
0 PAP

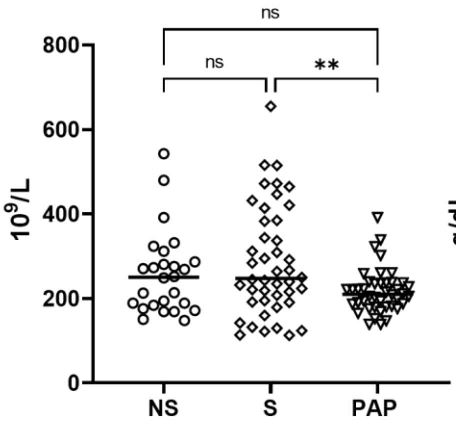

Hemoglobin

Number of subjects outer the reference range (>16 g/dL):
O NS
2 S (5%)
3 PAP (7%)

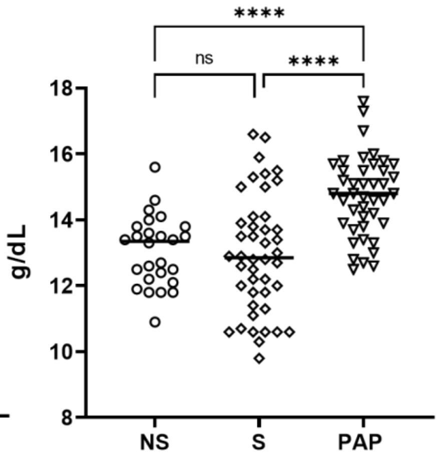

Figure 1S. Associations of NETs markers with biochemical parameters. Correlation matrix of NETs markers concentrations (NE. MPO, cir-nDNA, cir-mtDNA, MNR) with biochemical parameters for non-severe (A), severe (B), post-acute phase (PAP) (C) and all (D) COVID-19 patients. Heatmap manifests the strength of the relationship by Pearson's correlation analysis (red: positive correlation; blue: negative correlation).

A

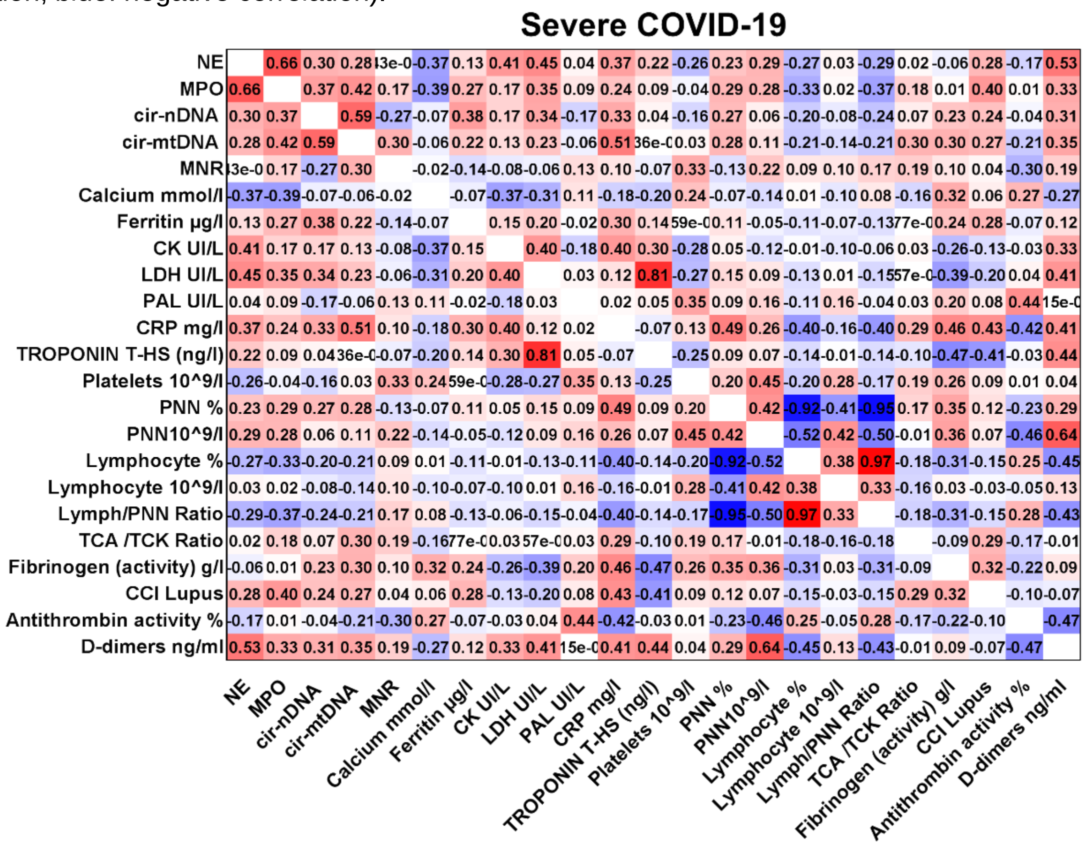

B

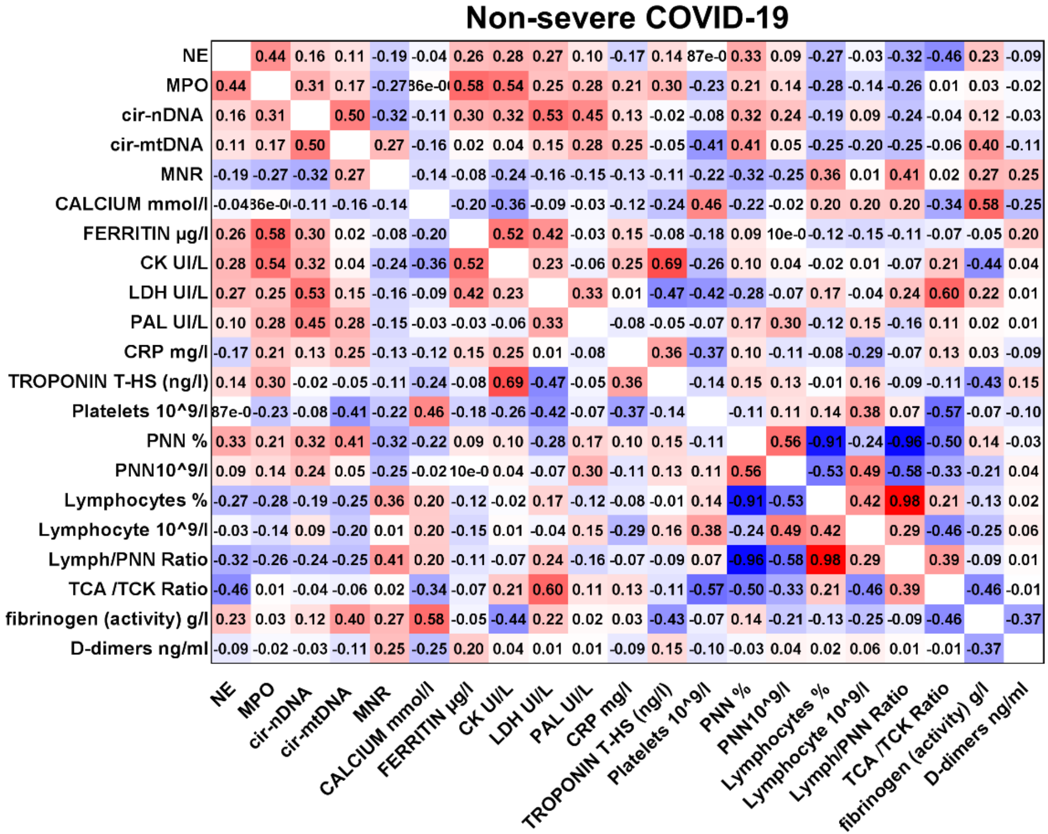

C

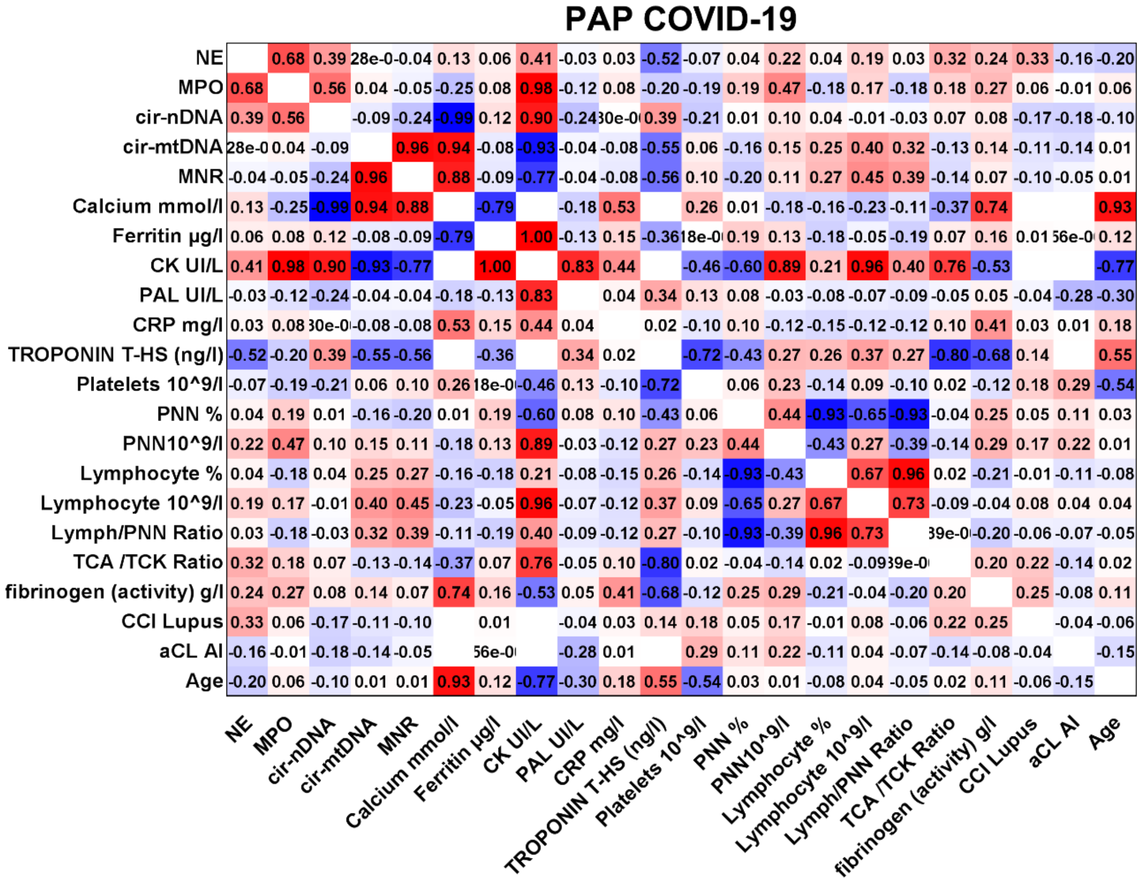
PAP COVID-19
NE
MPO
cir-nDNA
cir-mtDNA
MNR
Calcium mmol/l
Ferritin µg/l
CK UI/L
PAL UI/L
CRP mg/l
TROPONIN T-HS (ng/l)
Platelets 10^9/l
PNN %
PNN10^9/l
Lymphocyte %
Lymphocyte 10^9/l
Lymph/PNN Ratio
TCA /TCK Ratio
fibrinogen (activity) g/l
CCI Lupus
aCL AI
Age

D

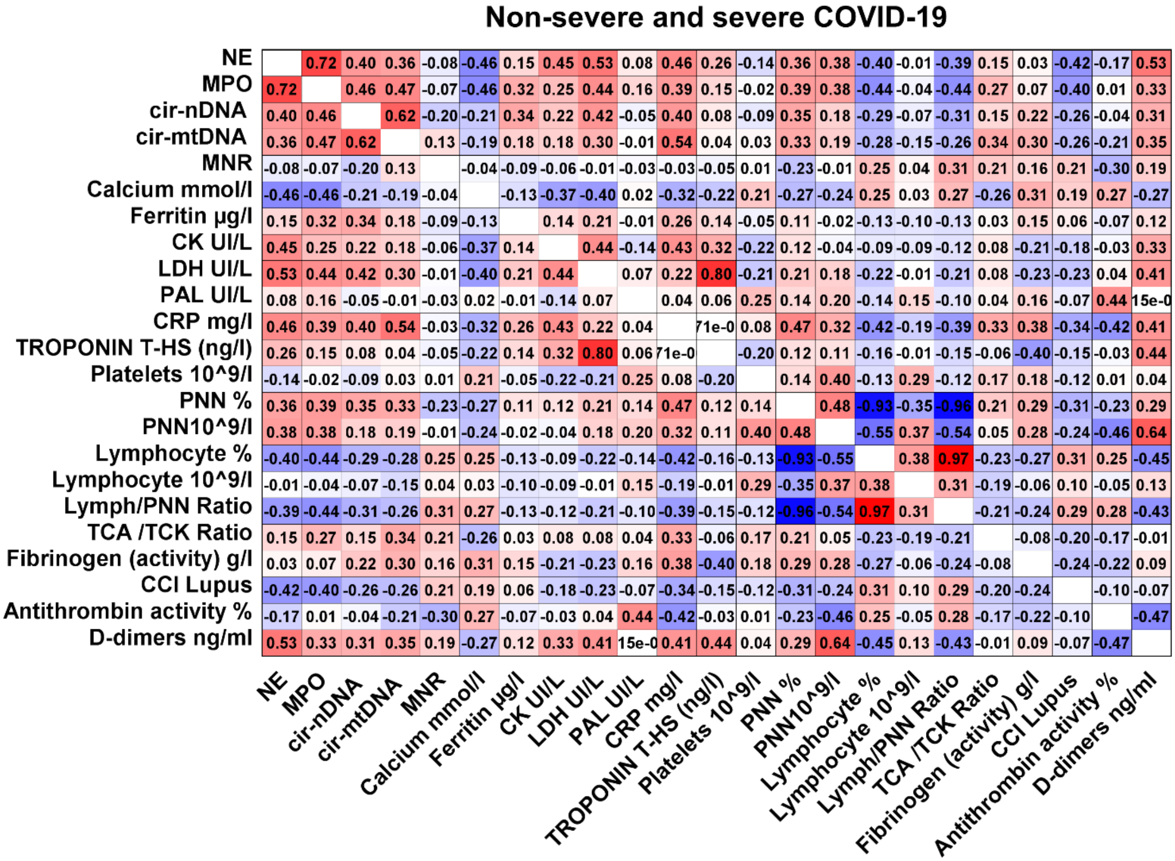
Non-severe and severe COVID-19
NE
MPO
cir-nDNA
cir-mtDNA
MNR
Calcium mmol/l
Ferritin µg/l
CK UI/L
LDH UI/L
PAL UI/L
CRP mg/l
TROPONIN T-HS (ng/l)
Platelets 10^9/l
PNN %
PNN10^9/l
Lymphocyte %
Lymphocyte 10^9/l
Lymph/PNN Ratio
TCA /TCK Ratio
Fibrinogen (activity) g/l
CCI Lupus
Antithrombin activity %
D-dimers ng/ml

Figure 2S. NETs markers normalized by PNN for COVID-19 patients and healthy individuals. (A) Comparison of NETs biomarkers in healthy, non-severe, severe and PAP COVID-19 cohorts. The values are medians with upper and lower limits. Note, for clarity P values level when comparing with HI are omitted but they are all significant vs NS, S and PAP for each biomarker; (B) Associations of NETs biomarkers in non-severe and severe COVID-19 patients and PAP cohort. ***The conclusions drawn on the association of NET biomarkers with disease severity do not depend on the number of polymorphonuclear neutrophils (PNN).***

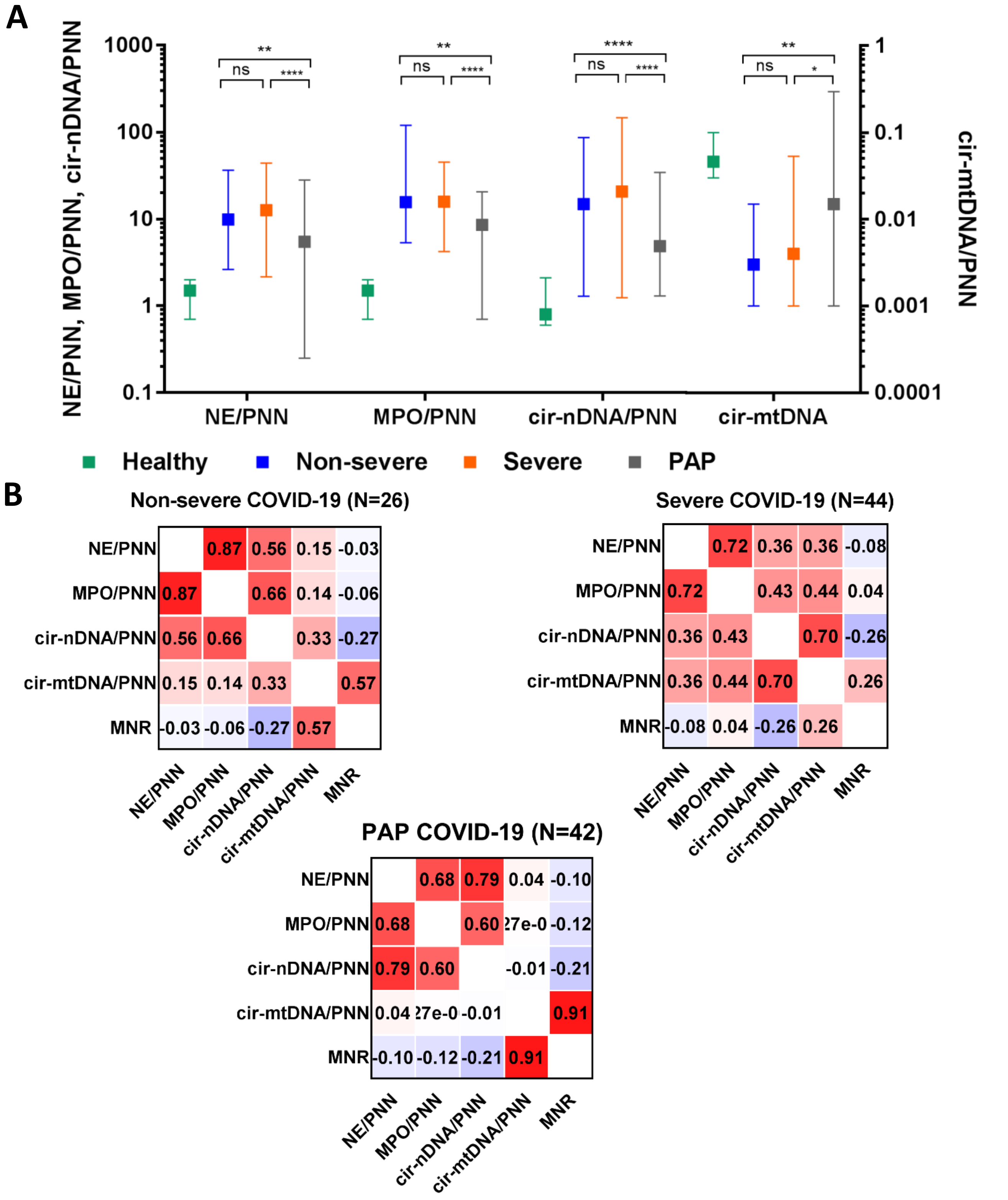

Figure 3S. NETs markers for COVID-19 patients and healthy individuals. There are individual values with medians for NE, MPO, cir-nDNA (A) and cir-mtDNA, MNR (B). «COVID-19 all» group is combination of non-severe and severe COVID-19 patients. «COVID-19 all» group's values for NETs biomarkers are between non-severe and severe COVID-19 cohorts.

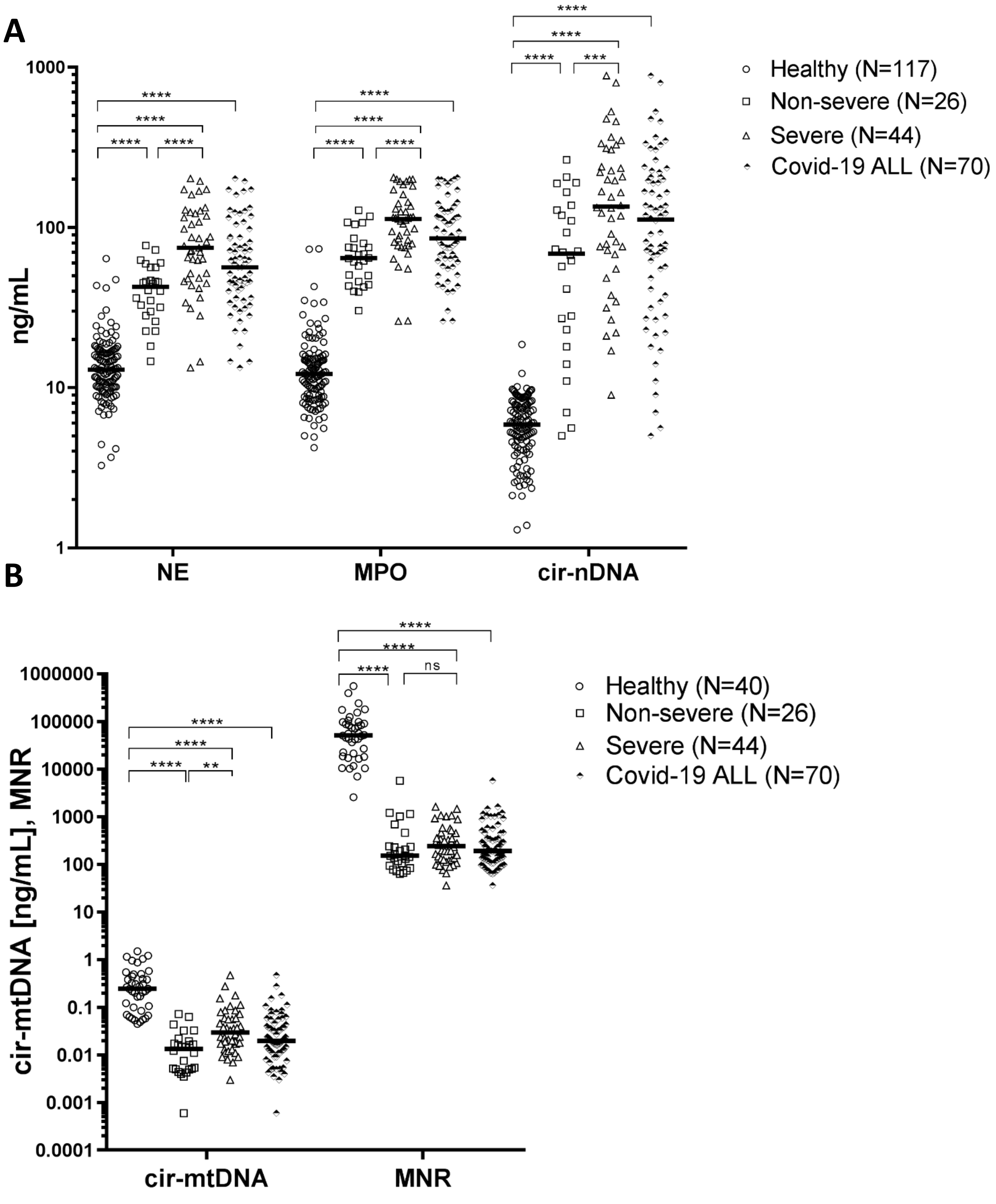

Figure 4S. ROC for NETs markers.

Performance characteristics of the NETs biomarkers for COVID-19. ROC curves for NE, MPO, cir-nDNA, cir-mtDNA concentrations and MNR between healthy individuals and COVID-19 patients (combining both NS and S COVID-19 patient cohorts). ROC: receiver operating characteristics; AUC: area under curve; NE: neutrophil elastase; MPO: myeloperoxidase; cirDNA: circulating cell-free DNA; cir-mtDNA: circulating cell-free DNA of mitochondrial origin; MNR: ratio of mitochondrial to nuclear circulating DNA concentration.

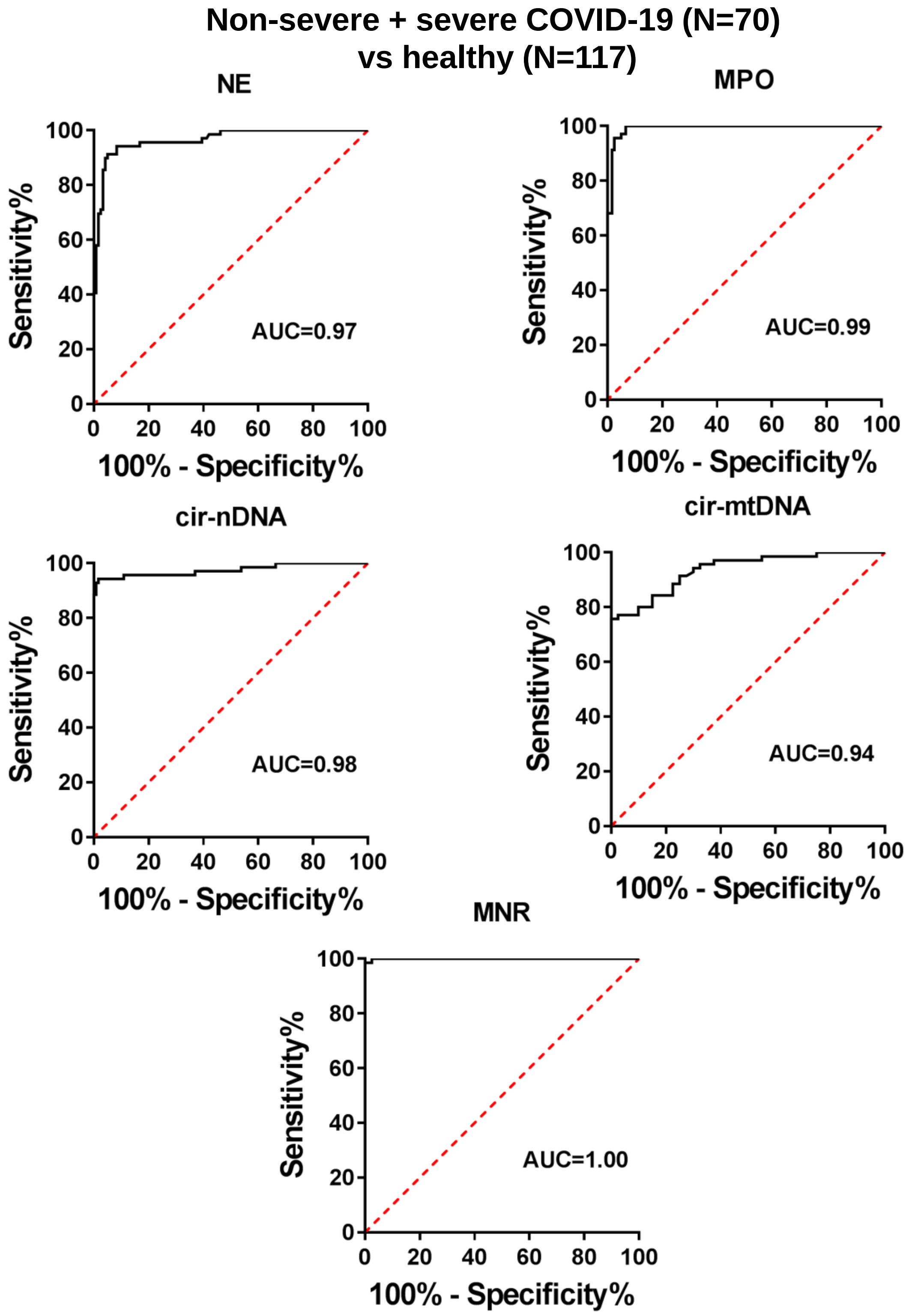

Figure 5S. ROC for NETs markers.

Performance characteristics of the NETs biomarkers for COVID-19. ROC curves for NE, MPO, combined NE+MPO, cir-nDNA, cir-mtDNA concentrations and MNR between non-severe and severe COVID-19 patients. ROC: receiver operating characteristics; AUC: area under curve; NE: neutrophil elastase; MPO: myeloperoxidase; cirDNA: circulating cell-free DNA; cir-mtDNA: circulating cell-free DNA of mitochondrial origin; MNR: ratio of mitochondrial to nuclear circulating DNA concentration.

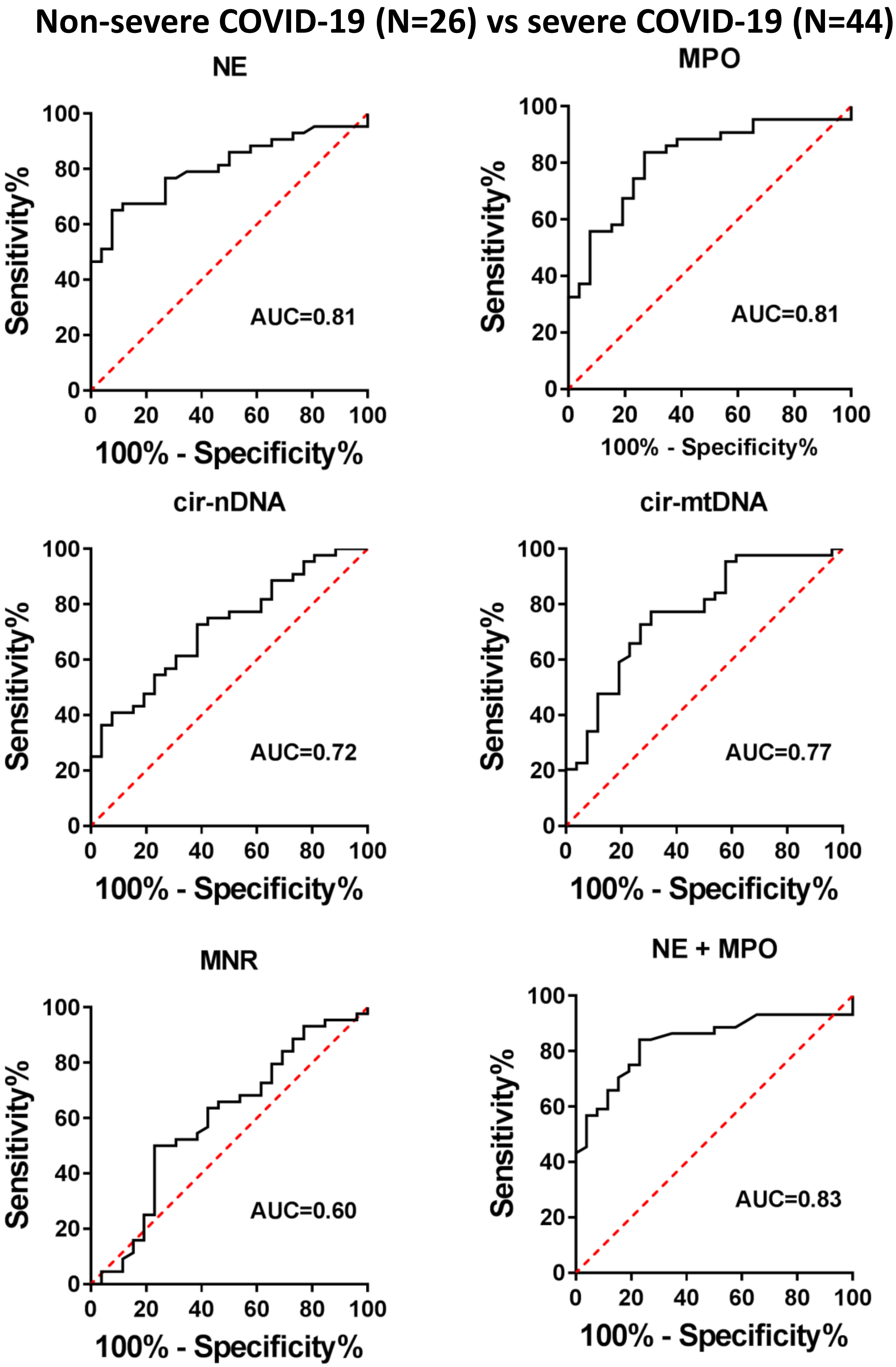

Figure 6S. ROC for NETs markers (NE+MPO).
Performance characteristics of the NETs biomarkers for COVID-19. ROC curves for combined NE+MPO between healthy individuals and non-severe, severe, post-acute (PAP) COVID-19 patients. ROC: receiver operating characteristics; AUC: area under curve; NE: neutrophil elastase; MPO: myeloperoxidase; cirDNA: circulating cell-free DNA; cir-mtDNA: circulating cell-free DNA of mitochondrial origin; MNR: ratio of mitochondrial to nuclear circulating DNA concentration.

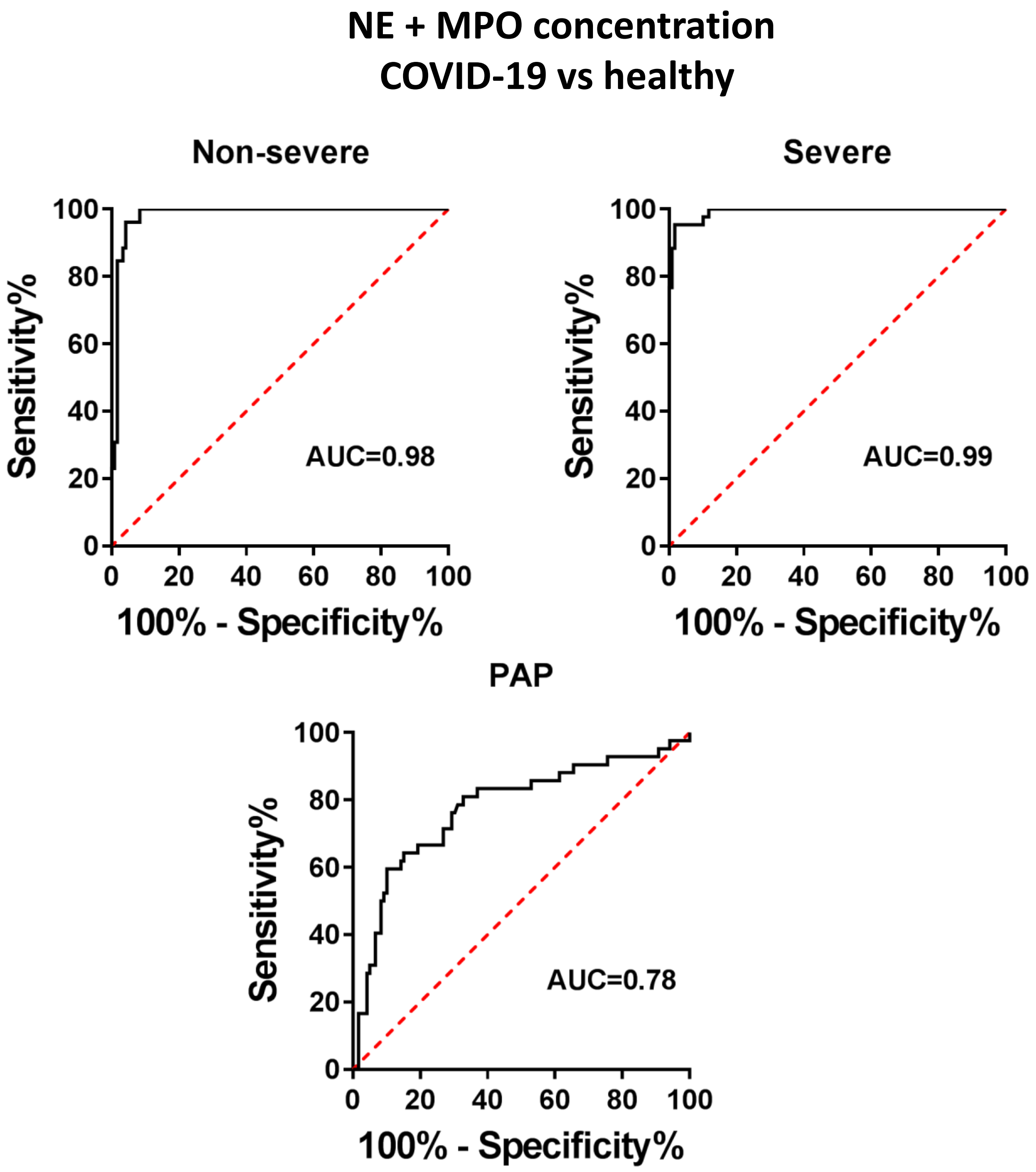

Figure 7S. Prevalence and number of patients positive to aCL and anti-B2GP autoantibodies in the NS, S and PAP cohorts

N	aCL IgG	aCL IgM	aCL IgG + IgM	antiB2GP IgG	antiB2GP IgM	antiB2GP IgG + IgM	Both aCL and antiB2GP	aCL or antiB2GP
Non-severe (N=18)	1	6	6	1	1	2	1	7
Severe (N=39)	5	7	9	3	4	4	4	9
Post-acute phase (N=42)	4	4	6	3	3	5	3	8
**%**	**aCL IgG**	**aCL IgM**	**aCL IgG + IgM**	**antiB2GP IgG**	**antiB2GP IgM**	**antiB2GP IgG + IgM**	**Both aCL and antiB2GP**	**aCL or antiB2GP**
Non-severe (N=18)	5.6	33.3	33.3	5.6	5.6	11.1	5.6	38.9
Severe (N=39)	12.8	17.9	23.1	7.7	10.3	10.3	10.3	23.1
Post-acute phase (N=42)	9.5	9.5	14.3	7.1	7.1	11.9	7.1	19
