# Supplementary material for: Persistence of neutrophil extracellular traps and anticardiolipin auto‐antibodies in post‐acute phase COVID‐19 patients
Source: J Med Virol. 2022 Oct 31;95(1):e28209. doi: 10.1002/jmv.28209 (PMC9874393; doi:10.1002/jmv.28209)
Supplement: Supplementary file 2 — Supplementary information. [file JMV-95-e28209-s002.docx]

**Supplement 1**

**Methods**

**Study population**

Patients hospitalized for COVID-19 infection

All consecutive patients above 18 years old hospitalized in the Intensive Care Unit (ICU) and in the Department of Infectious Diseases of the Lapeyronie Hospital (CHU Montpellier, France) between February and March 2021 with confirmed SARS-CoV2 infection, as determined by a positive result of a reverse transcription polymerase chain reaction (RT-PCR), were enrolled in this observational study. This study was approved by the local ethics committee (ID_202100715).

Patients were categorized as Severe vs Non-Severe depending on whether or not they met one or more of the following criteria: need for high flow nasal oxygen therapy (Optiflow; O2 > 15L/min) or mechanical ventilation, transfer to the ICU during the hospitalization, or occurrence of death.

The non-severe group consisted in 26 hospitalized subjects for COVID-19 infection because of hypoxemia or the presence of comorbidities All patients benefited from supportive care, such as the administration of oxygen and low molecular weight heparin to prevent thromboembolism. Most patients received 6 mg of intravenous dexamethasone once a day for at least 5 days, as recommended by the French High Council for Public Health (HCSP) on October 19th, 2020. No patients required transfer to an ICU, or died.

The Severe group consisted of 44 subjects hospitalized for COVID-19 in an ICU, according to local guidelines regarding ICU admission, the standard of care (including sedation, and neuromuscular blockade), the use of non-invasive ventilation, antibiotic treatment, as well as the use of rescue therapies for refractory hypoxemia (prone positioning, extracorporeal membrane oxygenation (ECMO), etc.). Initial oxygenation management included standard oxygen or high-flow nasal oxygen whenever needed. In the case of persistent hypoxemia, patients were sedated, intubated and mechanically ventilated according to current recommendations. In the case of refractory hypoxemia, prone position invasive ventilation was performed and, if unsuccessful, the patient underwent ECMO. Dexamethazone was administered at 6 mg intravenously daily for 10 days. Prophylactic anticoagulation was used for all patients.

Therapies instituted during ICU stay, including vasoactive drugs, invasive mechanical ventilation, renal replacement therapy and extracorporeal membrane oxygenation (ECMO), were recorded.

Patient consultation for post-COVID-19 follow-up

This group of Post-Acute Phase (PAP) COVID-19 patients consisted of 42 subjects previously hospitalized in an ICU, who were offered longitudinal monitoring 6 months or more after discharge, regardless of whether or not they were judged to have returned to full health. The presence or absence of post-COVID-19 complications was registered during medical follow-up, in parallel with additional blood tests performed with patients’ signed consent.

Among the 279 plasma from COVID-19 patients and HI individuals enrolled in the study, 229 (26 S, 44 NS, 42 PAP, and 117 HI) passed the quality control step and were subsequently analyzed (Fig 1).

**Data collection**

At admittance or consultation, the presence of comorbidities such as diabetes, hypertension, cardiovascular disease, chronic kidney disease (CKD), chronic obstructive pulmonary disease (COPD), cancer, chronic immunosuppression, dyslipidemia and transplantation, as well as current smoking status, was registered in the patient’s medical chart (Tables 1-2S in Supplement 2).

**Clinical laboratory analysis**

All hospitalized patients underwent biochemical and hematological explorations on venous blood sampled at admittance (Table 3S in Supplement 2). The same tests were performed on patients who underwent PAP follow-up (Table 4S in Supplement 2).

Following the routine laboratory procedure, biochemical analytes were measured according to an eGFR calculation based on levels of CFD-EPI creatinine, albumin, calcium, phosphate, ferritin, aspartate aminotransferase (ASAT), alanine aminotransferase (ALAT), alcaline phosphatase (ALP), gammaglutamyltransferase (GGT), lactate dehydrogenase (LDH), C-Reactive protein (CRP) (Cobas 8000, Roche®), procalcitonin (PCT) (Kryptor, ThermoFisher®) and glycated hemoglobin (HbA1c) (G8, Tosoh®). EDTA samples dedicated for HbA1c determination was centrifuged for 10 minutes at 4°C at 1200g immediately after use, and plasma stored at -80°C. Samples which could not be processed within 4 hours of collection were not analyzed.

Hematological analytes determination included leukocyte, neutrophil, thrombocyte, and lymphocyte counts (Pentra DX120, Horiba Medical), in addition to fibrinogen and D-Dimers (ACL TOP 700, Werfen®).

SARS-CoV2 infection was confirmed by a positive result of an RT-PCR assay on nasopharyngeal, pharyngeal swabs, or a lower respiratory tract aspirate sample. The presence of SARS-CoV2 variants was assessed by search of lineage B.1.1.7 (20I/501Y.V1 variant), B.13.5.1 (20H/501Y.V2 variant) and P.1 (20J/501Y.V3 variant) (SARS-CoV-2 / UK / SA Variant Triplex, ID Solutions). The absence of these three lineages was considered as the absence of any SARS-CoV2 variant.

**Healthy individuals**

We analyzed 114 healthy individuals (HI, 59 men and 55 women) from the Etablissement Français du Sang (EFS), which is Montpellier’s blood transfusion center (Convention EFS-PM N° 21PLER2015-0013). These samples were initially screened (virology, serology, immunology, blood numeration) and ruled out whenever any abnormality was detected.

**Blood collection and plasma isolation**

Samples were handled according to a pre-analytical guideline previously established by our group (Meddeb et al., 2019b). Blood samples from HI or patients were collected in 6-ml EDTA tubes (K2E, REF 367864, BD Vacutainer). The blood was then centrifuged at 1200 g at 4°C for 10 minutes. The supernatants were isolated in sterile 1.5 mL Eppendorf tubes and stored at -80°C. Plasma samples were then centrifuged at 16000 g at 4°C for 10 minutes (Mouliere et al., 2011). The plasma was distributed by 1.1 ml to the 1.5 ml Eppendorf tubes. Afterwards, the plasma was stored at -20°C until cirDNA extraction or ELISA analysis.

**DNA extraction from plasma**

CirDNA from plasma, serum or cell medium was extracted using the QIAmp DNA Mini Blood kit (Qiagen, Cat. No. 51104), according to the ‘‘Blood and body fluid protocol’’, using 80 µl of elution volume. DNA extracts were stored at -20°C until used.

**Quantification of DNA by qPCR**

The concentration of cirDNA was assessed using an integrated qPCR system that targets sequences of 67 bp and 320 bp size within the same region of the KRAS gene for cir-nDNA quantification, and of 67 bp in the mitochondrial Cytochrome oxidase III gene (MT-CO3) for cir-mtDNA quantification. A DNA integrity index (DII) was determined by calculating the ratio between the concentrations obtained with the primer pairs targeting long (320 bp) sequences unable to amplify those specific to the mononucleosome (size~180 bp) and short sequences (67 bp) that amplified all DNA fragments, including those associated to mononucleosomes (mono-N DNA). Q-PCR amplifications were carried out at least in duplicate in a 25 µL reaction volume on a CFX96 instrument using the CFX manager software (Bio-Rad). Each PCR reaction mixture was composed of 12.5 ml PCR mix (Bio-Rad Sso advanced mix SYBR Green), 2.5 ml of each amplification primer (0.3pmol/ml, final concentration), 2.5 ml PCR-analyzed water, and 5 ml DNA extract. Thermal cycling consisted of three repeated steps: a 3-min Hot-start Polymerase activation and denaturation step at 95 °C, followed by 40 repeated cycles at 95 °C for 10 s, and then at 60 °C for 30 s. Melting curves were obtained by increasing the temperature from 55 °C to 90 °C with a plate reading every 0.2 °C. The concentration was calculated from Cq detected by Q-PCR and a control standard curve on genomic DNA of known concentration and copy number. Serial dilutions of genomic DNA from human placenta (G1471, Promega) were used as a standard for quantification and their concentration. The MNR was calculated as [cir-mtDNA]/[cir-nDNA], where cir-mtDNA and cir-nDNA concentrations used in copies/ml of plasma.

**Human Myeloperoxidase (MPO) and Neutrophil Elastase (NE) quantifications**

MPO and NE concentrations were measured using enzyme-linked immunosorbent assay (ELISA) according to the manufacturer's standard protocol (Duoset R&D Systems, DY008, DY3174, and DY9167-05). Briefly, captured antibodies were diluted at the working concentrations in the Reagent Diluent (RD) provided on ancillary reagent kits (DY008), and coated overnight at room temperature (RT) on 96-well microplates with 100 μL per well. Then, captured antibodies were removed from the microplates, and wells were washed three times with 300 μL of Wash Buffer (WB). Microplates were blocked at RT for 2 hours by adding 300 μL of RD to each well. RD were removed from the microplates, and wells were washed three times with 300 μL of WB. Then, 100 μL of negative controls, standards and plasma samples (diluted 1/10) were added to the appropriate wells for one hour at RT. Samples, controls and standards were removed from the microplates, and wells were washed three times with 300 μL of WB. Detection antibodies were diluted at the working concentrations in the RD, and then added by 100 μL per well, for one hour at RT. Detection antibodies were removed from the microplates, and wells were washed three times with 300 μL of WB. Then, 100 μL of Streptavidin-HRP was added to each well and microplates were incubated at RT for 30 minutes. Repeat wash three times. Finally, 100 μL per well of substrate solution was added and incubated for 15 minutes, and the Optical Density (O.D) of each well was read immediately at 450 nm with the PHERAstar FS instrument using the PHERAstar control software.

**Anti-cardiolipin antibodies**

The anti-cardiolipin antibodies (aCL, IgG/IgM) were assessed using the Orgentec ELISA kit. Quantitative assessment of aCL was considered for IgG or IgM >= 3 UA/ml (the median in negative individuals is 1 UA/ml using this test). aCL are considered to be the predominant antigen in APS. The Beta-2-glycoprotein I antibody (anti-B2GPI) binds anionic phospholipids such as aCL, and anti-B2GPI are recognized in the laboratory as criteria for APS diagnosis. It is well known that anticardiolipin antibodies can be detected in conditions other than APS. Medications, infections and other illnesses

have been reported in association with aCL, which are often transient and unsustained. While less is known about the associations and significance of anti-B2GPI outside of APS, several studies have addressed this issue in selected patient populations with diseases other than APS. An increased prevalence of anti-B2GPI was reported in several infections (HIV, syphilis, malaria, leprosy and hepatitis C), as well as in a variety of disorders such as autoimmune hepatitis, coeliac disease, metabolic syndrome, and hemodialysed patients with end stage renal failure (Anna Brusch, doi:10.3390/antib5020016). However, in some circumstances the presence of anti-B2GPI may confer a worse prognosis of the underlying disease, for instance, in the case of end stage renal failure patients receiving haemodialysis. The rather low aCL test specificity is improved by the use of the ELISA as a confirmatory, specific test for B2GPI antibodies, usually being performed after a positive screening test result for aCL. Quantitative assessment of aCL antibodies was considered for IgG or IgM >= 3 UA/ml (upon the 99th percentile as reported by Montaruli et al, Rheumatology, 2012, using the Orgentec Diagnostika Elisa kit.). Quantitative assessment of anti-B2GP antibodies was considered for IgG >= 4 UA/ml and IgG>= 5 UA/ml (upon the 90th percentile as reported by Vikerfors et al (Lupus, 2012). Note, the Orgentec Diagnostika kit propose a cutoff of 30 and 40 for stringently diagnosing APS.

**Statistical Analysis**

Statistical analysis was performed using the GraphPad Prism V6.01 software. Correlation analysis was performed using the Pearson test. The Mann-Whitney test was used to compare means. A probability of less than 0.05 was considered to be statistically significant; *p<0.05, **p<0.01; ***p< 0.001; ****p< 0.0001.
